# Association between Vitamin D deficiency and Breast Cancer

**DOI:** 10.12669/pjms.333.11753

**Published:** 2017

**Authors:** Noureen Shaukat, Farhat Jaleel, Foad Ali Moosa, Naeem Akhter Qureshi

**Affiliations:** 1 Dr. Noureen Shaukat, MBBS. Department of Surgery, Dow University of Health Sciences, Karachi, Pakistan; 2 Dr. Farhat Jaleel, MBBS, FCPS. Associate Professor, Department of Surgery, Dow University of Health Sciences, Karachi, Pakistan; 3 Prof. Dr. Foad Ali Moosa, MBBS, FRCS. Department of Surgery, Dow University of Health Sciences, Karachi, Pakistan; 4 Dr. Naeem Akhter Qureshi, MBBS, FCPS. Assistant Professor, Department of Surgery, Dow University of Health Sciences, Karachi, Pakistan

**Keywords:** Breast cancer, Vitamin D deficiency

## Abstract

**Objective:**

To determine the association between vitamin D deficiency and breast cancer.

**Methods:**

This case control study included 94 female patients aged 20-75 years of any marital status and parity. Newly diagnosed 42 breast cancer patients who presented to surgical OPD of Dow University Hospital from Jan 2016 to June 2016 were included into the study as “cases” after informed consent. Age-matched 52 females who presented to OPD for complain other than breast pathology were included as the “control group”. The sociodemographic of both cases and controls and histopathological characteristics of cases were recorded. Serum 25-(OH)2D levels were studied by the ELISA technique and recorded in ng/ml. Vitamin D deficiency was considered at serum level less than 20 ng/ml.

**Results:**

Mean age was 40.1 Years for controls and 47.6 Years for cases. Mean height, weight and BMI did not differ between cases and controls. Serum Vitamin D levels were significantly lower in cases (85.7%) than controls (55.8%). The unadjusted and adjusted ORs for breast cancer in cases and controls showed a statistically significantly increased risk of breast cancer with low vitamin D concentration (p value0.003). After adjustment for age, parity, BMI, sun exposure, economic status and education status the ORs (95% CIs) for breast cancer risk was7.8 (1.99 - 30.58) for women with vitamin D concentrations <20 ng/mL.

**Conclusion:**

Findings of our study conclude that vitamin D deficiency is associated with risk of breast cancer.

## INTRODUCTION

Breast cancer poses a serious health risk for women throughout the world. It is the most common cancer among women in United States.^1^ It accounts for 22% of all female cancers worldwide and approximately 42% cases occur in developing countries.^2^ Breast cancer is also the commonest cancer of females in Southern and Northern Pakistan.^2^

For the large magnitude of breast cancer, a lot of efforts are being made to identify such risk factors that can be modified to prevent breast cancer. Vitamin D is assumed to be one of such risk factors. It is a fat soluble vitamin. Its main sources are skin production (via exposure to ultraviolet light) and dietary intake. Vitamin D has an important role in calcium and bone homeostasis. Vitamin D has not only shown to strengthen our bones but it also has capability to modulate several features of cancer. Its anticarcinogenic properties include inhibition of cell proliferation, invasion, metastasis and angiogenesis and induction of apoptosis and differentiation.^3^

In a study done in Shaukat Khanum Memorial Cancer Hospital and Research Centre, Lahore, Pakistan vitamin D deficiency was found in 95.6% of breast cancer patients and in 77% of control group.^4^

Several studies have been done to evaluate the association of vitamin D deficiency and breast cancer risk. There is controversy in the literature about this association. Some studies have found that breast cancer is associated with low levels of vitamin D.^5^-^9^ However there is a study published in 2007 that showed no association between breast cancer risk and vitamin D levels.^10^ Low vitamin D levels is frequently found in our population.^11^ If we can establish an association between the two in our population we will be able to give recommendations to take corrective actions and try to reduce the incidence of breast cancer in our population.

## METHODS

This case control study was approved by the College of Physicians and Surgeons Pakistan. Sample size was calculated by using WHO sample size calculator with 80% power of test and 5% level of significance considering proportions (P1=95.6%)^4^ and (P2=77%)^4^ in case and control groups to beatleast42 patients in each group. Newly diagnosed 42 breast cancer patients who presented to the OPD of surgical department of Dow University Hospital were included into the study as “cases” after informed consent over a period of 6 months from Jan 2016 to June 2016. Age-matched 52 females who presented to OPD for complain other than breast pathology were recruited as the “control group.” Patients were recruited according to non-probability consecutive, meeting inclusion and exclusion criteria.

### Inclusion criteria

1. Age 20-75 years 2. Resident of Karachi for >5 years (confirmed by seeing NIC) Cases: Diagnosed with invasive breast cancer within 6 months of all grades and of stage I-III.

### Controls

Age matched females presenting in clinic with no breast pathology.

### Exclusion criteria

1. Pregnant 2. Lactating 3. Males 4. Stage IV breast cancer 5.Patients receiving neoadjuvant chemotherapy. The sociodemographic were recorded by direct questioning on to the proforma for the whole study population. The histopathological diagnosis of breast cancer, grade, stage of the tumor, and hormone receptor status (estrogen receptor - ER, progesterone receptor-PR, and Her2neu) was recorded from the pathology reports of breast cancer patients. Serum 25-(OH)2D levels were studied by the ELISA technique on the blood samples drawn of the study population at their initial presentation and the values were recorded in ng/ml. Vitamin D deficiency was considered at serum level less than 20 ng/ml.

### Statistical Analysis

Data was analyzed by using SPSS version 20 on computer. Categorical variables in cases and controls like marital status, parity, grades, stages, histopathology, receptor status of tumor, exposure to sunlight, economic status, education status and vitamin D deficiency were presented in frequency and percentages. Mean+/-SD was computed for numerical variables like age, size of tumor, duration of carcinoma breast, height, weight and BMI. For comparing baseline characteristics, P-values were calculated with Chi-square analyses for categorical variables and Mann-Whitney test for numerical variables. A P-value of ≤0.05 was considered statistically significant. Logistic regression modeling was used for assessment of the association between vitamin D deficiency and risk of breast cancer. Potentially confounding variables that were evaluated were age, parity, BMI, sun exposure, education and economic status. P value ≤0.05 was taken as significant.

## RESULTS

Data of 94 women, including 42 breast cancer cases and 52 controls, were included in analysis. The characteristics of study population are shown in Table-I and II. Mean age was 43.47 years, mean height was 1.54 meters, mean weight 68.01 kilograms, mean BMI 28.83 kg/m^2^, mean duration was 6.14 months and mean size of breast cancer was 3.82 centimeters. Mean age was 40.1 year for controls and 47.6 year for cases. Mean height, weight and BMI did not differ between cases and controls.

**Table-I:** Descriptive of all study variables.

*Group*	*N (%)*
Control	52 (55.3)
Case	42 (44.7)
***Vitamin D deficiency***
Yes	65 (69.1)
No	29 (30.9)
***Age Group***
<40 years	33 (35.1)
≥40 years	61 (64.9)
***BMI***
Underweight	14 (14.9)
Overweight	54 (57.4)
Obese	26 (27.7)
***Parity***
≤2	45 (47,9)
≥3	49 (52.1)
***Sun exposure***
<20min/day for 2 yrs	60 (63.8)
20-30min/day for 2 yrs	18 (19.1)
>30min/day for 2 yrs	16 (17)
***Economic status***
Family monthly income < Rs.5000	12 (12.8)
Family monthly income Rs.5000-10,000	18 (19.1)
Family monthly income > Rs.10,000	64 (68.1)
***Education status***
Illiterate	21 (22.3)
Primary	11 (11.7)
Matric	25 (26.6)
Inter and above	37 (39.4)

BMI: Body Mass Index

**Table-II:** Descriptive of continuous variables.

	*Control Mean (SD)*	*Case Mean (SD)*	P-value*
Age (y)	40.11 (12.7)	47.6 (12.1)	0.004
Height (m)	1.53 (0.10)	1.54 (0.07)	0.908
Weight (kg)	68.00 (13.6)	68.02 (8.2)	0.822
BMI (kg/m^2^)	28.85 (6.14)	28.80 (5.39)	0.867

* p-value was calculated using Mann-Whitney test.

Serum Vitamin D levels were significantly lowers in cases than controls. 85.7% of cases and 55.8% of controls had Vitamin D levels less than 20 ng/ml. Only 14.3% of cases and 44.2% of controls had Vitamin D levels above 20 ng/ml (Table-III).

**Table-III:** Association of all study variables among cases and controls.

	*Occurrence of Breast cancer*		*P-value*

	*Control n (%)*	*Case n (%)*
***Vitamin D***			
Yes	29 (55.8)	36 (85.7)	0.002
No	23 (44.2)	6 (14.3)	
***Marital Status***			
Married	50 (96.2)	42 (100)	0.119
Unmarried	2 (3.8)		
***Age group***			
<40 years	24 (46.2)	9 (21.4)	0.013
≥40 years	28 (53.8)	33 (78.6)	
***BMI***			
Underweight/normal	9 (17.3)	5 (11.9)	0.042
Overweight	24 (46.2)	30 (71.4)	
Obese	19 (36.5)	7 (16.7)	
***Parity***			
≤2	26 (50)	19 (45.2)	0.646
≥3	26 (50)	23 (54.8)	
***Sun Exposure***			
<20min/day for 2 yrs	31 (59.6)	29 (69.0)	0.531
20-30min/day for 2 yrs	12 (23.1)	6 (14.3)	
>30min/day for 2 yrs	9 (17.3)	7 (16.7)	
***Economic Status***			
Family monthly income <5000Rs	6 (11.5)	6 (14.3)	0.275
Family monthly income 5000-10000Rs	13 (25.0)	5 (11.9)	
Family monthly income >10000Rs	33 (63.5)	31 (73.8)	
***Education Status***			
Illiterate	16 (30.8)	5 (11.9)	0.178
Primary	5 (9.6)	6 (14.3)	
Matric	13 (25.0)	12 (28.6)	
Inter and above	18 (34.6)	19 (45.2)	

BMI: Body Mass Index, *p-value was calculated using Chi-square test.

The ORs for breast cancer in cases and controls by category of vitamin D status are shown in Table-IV. The unadjusted and adjusted ORs showed a statistically significantly increased risk of breast cancer with low vitamin D concentration (OR 4.75 95% CI 1.71-13.23; p-value0.003). After adjustment for age, parity, BMI, sun exposure, economic status and education status, the risk for breast cancer was doubled (OR 7.83 95% CI 1.99-30.58; p-value0.003) for women with vitamin D concentrations<20 ng/mL.

**Table-IV:** Factors associated with occurrence of Breast cancer, (n=94).

*Characteristics*	*Occurrence on Breast cancer*			

	*OR (95% CI)*	*p-value*	*AOR (95% CI)*	*p-value*
***Vitamin D deficiency***				
No	1		1	
Yes	4.75 (1.71-13.23)	0.003	7.83 (1.99-30.58)	0.003
***Age group***				
< 40 years	1		1	
≥ 40 years	3.14 (1.25-7.86)	0.014	6.63 (1.68-26.67)	0.007
***Parity***				
≤ 2	1		1	
≥3	1.21 (0.53-2.73)	0.646	1.02 (0.32-3.25)	0.963
***BMI***				
Underweight/normal	1		1	
Overweight	2.25 (0.66-7.60)	0.192	1.29 (0.23-7.02)	0.783
Obese	0.66 (0.16-2.67)	0.564	0.26 (0.04-1.69)	0.159
***Sun exposure***				
<20min/day for 2 yrs	1		1	
20-30min/day for 2 yrs	0.53 (0.17-1.61)	0.266	0.33 (0.08-1.37)	0.129
>30min/day for 2 yrs	0.83 (0.27-2.52)	0.744	2.24 (0.46-10.99)	0.313
***Economic status***				
Family monthly income <Rs.5000	1		1	
Family monthly income Rs.5000-10000	0.38 (0.08-1.77)	0.221	0.21 (0.02-1.60)	0.134
Family monthly income >Rs.10000	0.93 (0.27-3.22)	0.921	0.41 (0.07-2.17)	0.297
***Education status***				
Illiterate	1		1	
Primary	3.84 (0.81-18.17)	0.090	7.30 (0.93-56.97)	0.058
Metric	2.95 (0.82-10.56)	0.096	9.13 (1.43-58.20)	0.019
Inter and above	3.37 (1.02-11.14)	0.046	6.47 (1.07-38.90)	0.041

OR: crude odds ratio, CI: confidence intervals, AOR: adjusted odds ratio for all variables, BMI: Body mass index.

## DISCUSSION

Vitamin D deficiency is very common as shown in our results that 55.8% of controls and 88.7% of breast cancer patients are vitamin D deficient. This finding is slightly different from a study done in Shaukat Khanum Memorial Cancer Hospital and Research Centre Lahore, that showed vitamin D deficiency in 76.7% of controls and 95.6% of cases.^4^ However, there is marked difference in our findings as compared to a study of Crew et al that showed vitamin D deficiency (levels less than 20ng/ml) in 28% of controls and 33% of cases.^1^ Many observational and crossectional studies reflect that deficiency of vitamin D is commonly found in patients with breast cancer.^12^,^13^ Some studies have also demonstrated a protective effect of vitamin D on breast cancer risk.^14^ Several studies have shown a negative relationship between sunlight exposure and breast cancer risk. Proposed mechanism for this negative association is sunlight induced dermal synthesis of vitamin D, which evidence suggests, can reduce the risk of breast cancer.^15^

In our study, parity, BMI, sun exposure, economic status and education status did not show any statistically significant association with breast cancer risk. In our study 71.4% of cases and 46.2% of controls were overweight that may link increased frequency of vitamin D deficiency in cases as compared to controls because of high BMI as shown in study of Imtiaz et al.^4^ The topic of vitamin D deficiency and breast cancer risk is a field of intense study and many aspects of it require further investigations.

### Conclusion

Findings of this study show that vitamin D deficiency is associated with risk of breast cancer.

***Authors` contribution:***

**NS:** Conceived, designed, did data collection, statistical analysis, manuscript writing, revising critically, editing and final approval of manuscript.

**FJ:** Designed, did data collection, editing, revising critically and final approval of manuscript.

**FAM and NAQ:** Contributed in acquisition of data, revising the article critically and final approval of the article.
